# Prognostic influence of small leucine-rich proteoglycans on serous ovarian cancer

**DOI:** 10.1007/s00432-026-06529-2

**Published:** 2026-06-07

**Authors:** H. Surmann, A. Bartha, B. Győrffy, L. Kiesel, L. Hanker, M. Götte

**Affiliations:** 1 Department of Gynecology and Obstetrics, Münster University Hospital, Albert-Schweitzer-Campus 1, 48149 Münster, Germany; 2 Department of Bioinformatics, Semmelweis University, Budapest, Hungary; 3 Department of Biophysics, University of Pecs, Pecs, Hungary; 4 HUN-REN TTK Cancer Biomarker Research Group, Budapest, Hungary; 5 Cells-in-Motion Interfaculty Center (CiMIC), University of Münster, Münster, Germany

**Keywords:** Ovarian cancer, Proteoglycans, Prognosis, Chemotherapy

## Abstract

**Purpose:**

Ovarian cancer is one of the most lethal cancers in women worldwide. To be able to offer successful treatment and improve the prognosis, knowledge of factors influencing the tumor microenvironment is indispensable. In this context, the influence of the extracellular matrix on tumor progression is increasingly recognized. Of note, preclinical data in cell line and animal models have suggested that several members of the small leucine-rich proteoglycan (SLRP) family are mechanistically involved in the regulation of tumor progression. We hypothesized that dysregulation of SLRP expression may have a prognostic value in ovarian cancer.

**Methods:**

To distinguish whether this expression is altered in the cells themselves or in the extracellular matrix, quantitative Real-Time PCR was performed on ovarian cancer cell lines and complemented by analysis of CCLE datasets. We used Kaplan–Meier survival curves to investigate whether a high or low mRNA expression influences the survival of ovarian cancer patients. Finally, the interactions of the SLRPs were investigated using a STRING analysis.

**Results:**

We demonstrated the potential beneficial effect of a low mRNA expression of most SLRPs on the prognosis of serous ovarian cancer. STRING analysis revealed interactions with other proteins already known to influence tumor behavior and metastasis of various carcinomas.

**Conclusion:**

These findings suggest that SLRPs may be involved in ovarian cancer biology and could represent candidates for further mechanistic investigation. However, their potential relevance for therapeutic strategies, including treatment response, requires additional functional validation.

**Supplementary Information:**

The online version contains supplementary material available at 10.1007/s00432-026-06529-2.

## Objective

Ovarian cancer is one of the most lethal cancer types in women worldwide. It is the fifth most common cause of cancer death. Within gynecological tumors, ovarian cancer has the poorest prognosis and highest mortality (Roett and Evans 2009). Patients who are diagnosed with stage IV serous ovarian cancer have a five-year survival rate below 10% (Roett and Evans 2009). Current diagnostic procedures include thorough examination of the abdomen and pelvis, which is combimed with imaging by ultrasound or CT. Serum biomarkers such as antigen-125 (CA-125), carcinoembryonic antigen (CEA), and CA 19-9 complement this procedure, while histopathological investigation allows for distinguishing the clinical subtype (Redman et al. 2011; Penn and Alvarez 2023).

Personalized therapies are increasingly becoming the standard in all areas of medicine. They might also be an option for patients with ovarian cancer in the future. Identification of biomarkers could be significant to facilitate a prognosis and determine the appropriate therapy for each patient to reduce the mortality (Radu et al. 2021).

Small leucine-rich proteoglycans (SLRPs) are part of the extracellular matrix. Class 1 and 2 include Biglycan (BGN), Decorin (DCN), Asporin (ASPN), ECM2, ECMX, Fibromodulin (FMOD), Lumican (LUM), PRELP, Keratocan (KERA) and Osteomodulin (OMD). Their main function is the regulation of collagen synthesis (Iozzo and Schaefer 2015). Most tumors create a fibrotic area around them mainly consisting of collagen fibers and fibroblasts (Naito 2005). Further known functions are an influence on cell proliferation and inflammatory mechanisms (Appunni et al. 2019) as well as having an impact on angiogenesis, adhesion, and migration which are crucial factors for cancer progression and metastasis (Iozzo and Schaefer 2015). The function that SLRPs have in serous ovarian cancer has not been fully established yet.

It is important to understand the impact SLRPs have on cancer to improve therapy in the future The purpose of this work is to determine whether there is a prognostic influence of class 1 and 2 SLRPs on serous ovarian cancer.

## Materials and methods

### Gene expression analysis in tumor, normal, and metastatic tissue

TNM-Plot (https://tnmplot.com/analysis/) was used to compare the gene expression of SLRPs in different tissues. TNM-Plot is an open access online database that compares gene expression in tumor, normal, and metastatic tissues. The database uses gene-arrays from the Gene Expression Omnibus of the National Center for Biotechnology Information and RNA-sequencing from The Cancer Genome Atlas, Therapeutically Applicable Research to Generate Effective Treatments, and The Genotype-Tissue Expression (Bartha and Győrffy 2021).

“Compare Tumor, Normal and Metastasis” was used for the analysis. Analysis was performed for all class 1 and 2 SLRPs. The database did not include any data for ECMX. Statistical comparison was performed by Kruskal–Wallis and Dunn´s Test.

The database includes a total of 834 patients, consisting of 46 normal tissue samples, 744 tumor tissue samples, and 44 metastatic tissue samples. Staging information is available for for 405 patients. Among these, 73 are classified as Stage I, 37 as Stage II, 262 as Stage III, and 33 as Stage IV.

### Quantitative real time PCR

Quantitative Real Time PCR (qPCR) was used to quantify and compare the expression levels of SLRPs in established human ovarian cancer cell lines. The ovarian cancer cell lines that were used are SKOV3 (RRID:CVCL_0C84), CAOV3 (RRID:CVCL_C8XN), SW626 (RRID:CVCL_1725), and PA1 (RRID:CVCL_0479). The cells were purchased from ATCC, they correspond to the ‘Ovarian Cancer Panel’. CAOV-3, SW626 and PA-1 were maintained in DMEM containing 10% FCS, and 1% penicillin/streptomycin in a humidified atmosphere of 7.5% CO_2_ at 37 °C. SKOV-3 cells were maintained in McCoy’s 5A containing 10% FCS (fetal calf serum), 1% glutamine, and 1% penicillin/streptomycin in a humidified atmosphere of 5% CO_2_ at 37 °C.

Total RNA was isolated from the cells with the InnuPrep RNAMini Kit (Analytik Jena AG, Jena, Germany). RNA concentration and purity were assessed using an Eppendorf BioPhotometer (Eppendorf, Hamburg, Germany). Only samples with an A260/A280 ratio between 1.8 and 2.0 were considered suitable for further analysis. RNA was reverse transcribed into cDNA using the High-Capacity cDNA Reverse Transcription Kit (Applied Biosystems, Foster City, CA, USA). Using a Mastermix from Eurogentec (Eurogentec, Liège, Belgium) the qPCR was conducted. For gene expression detection the qPCR System ABI PRISM 7300 Sequence Detection System (Thermo Fisher Scientific, Waltham, USA) was used. The examined SLRPs were BGN, DCN, ASPN, LUM, PRELP and OMD. The primer sequences were from Biolegio (Nijmegen, Netherlands) and are listed in Supplementary Table S1. Actin was used as a housekeeping gene. Mean delta CT value, mean fold change and its standard deviation for all cell lines in comparison to SKOV3 were calculated using the 2^−delta delta Ct^ method (Livak and Schmittgen 2001). Statistical analysis was performed using GraphPad Prism (RRID:SCR_002798). Data distribution was assessed using the Shapiro–Wilk test. For comparisons across multiple groups, normally distributed data were analyzed using one-way ANOVA followed by Tukey’s post hoc test, whereas non-normally distributed data were analyzed using the Kruskal–Wallis test with Dunn’s multiple comparison correction. All experiments were performed in three independent biological replicates, each with technical triplicates. A *p*-value < 0.05 was considered statistically significant.

### Analysis of ovarian cell line gene expression data from the CCLE datasets

Using the Cancer Cell line Encyclopedia (CCLE) datasets (RRID:SCR_013836), gene expression data from 1019 cell lines were downloaded from the DepMap portal (Tsherniak et al. 2017). From these, we identified 43 ovarian cancer cell lines, comprising 13 metastatic, 29 primary tumor-derived, and 1 immortalized ovarian cell line. TPM-normalized expression values were used to generate heatmaps, constructed using the ComplexHeatmap R package (RRID:SCR_017270)(Gu et al. 2016). For statistical comparison between primary and metastatic cell lines, we performed the Mann–Whitney U test, with significance set at *p* < 0.05.

### Immunohistochemistry (Human Protein Atlas)

Immunohistochemical data were obtained from the Human Protein Atlas (RRID:SCR_006710; https://www.proteinatlas.org; version 21.0). Representative staining images for selected SLRPs (BGN, DCN, ASPN, ECM2, LUM, KERA) in serous ovarian carcinoma were retrieved from the pathology section of the database. The remaining class 1 and 2 SLRPs were unavailable in the database. The Human Protein Atlas provides standardized immunohistochemical staining performed on formalin-fixed, paraffin-embedded tissue sections using validated antibodies (Sjöstedt et al. 2020).

Representative tumor samples were selected to illustrate spatial distribution of protein expression within ovarian cancer tissue. The analysis focused on qualitative assessment of staining patterns, including localization and distribution of signal, rather than quantitative comparison of expression levels. Staining patterns were evaluated visually with particular attention to localization within tumor epithelial versus stromal and extracellular matrix compartments. Direct links to the individual immunohistochemical images retrieved from the Human Protein Atlas are provided in Supplementary Table S3 for transparency and reproducibility.

### Survival analysis

For survival analysis, the Kaplan–Meier-Plotter (RRID:SCR_024521; https://kmplot.com/analysis/) was used. The open-access online database has collected microarray gene expression data and clinical information on survival from NCBI-GEO and The Cancer Genome Atlas. Using the statistical program `R`, they have created Kaplan–Meier survival curves (Győrffy et al. 2012). The survival curves compared high expression with low expression of the selected genes. Hazard Ratio and Logrank P values were also computed for the selected settings. Median survival in months was calculated for each gene. Survival curves were plotted for each class 1 and 2 SLRP for the outcomes progression-free survival and overall survival. There was no data available for ECMX. The patients were split by median in high and low gene expression. The setting ‘only JetSet best probe set’ was used. *P*-values were adjusted for multiple testing using the Benjamini–Hochberg false discovery rate (FDR) method (Benjamini and Hochberg 1995). A level of significance of *p* < 0.05 was chosen.

### STRING analysis

STRING database (RRID:SCR_005223; https://string-db.org/) was used to investigate protein–protein interactions. The online database showes known and predicted interactions. An interaction network was generated using proteoglycans from the class 1 and 2 SLRP. ECMX was not available in the database. The results were selected by the high confidence threshold (0.700). The 1st and 2nd shell each showed no more than ten interactors. All available interaction sources were activated.

## Results

**Fig. 1:**
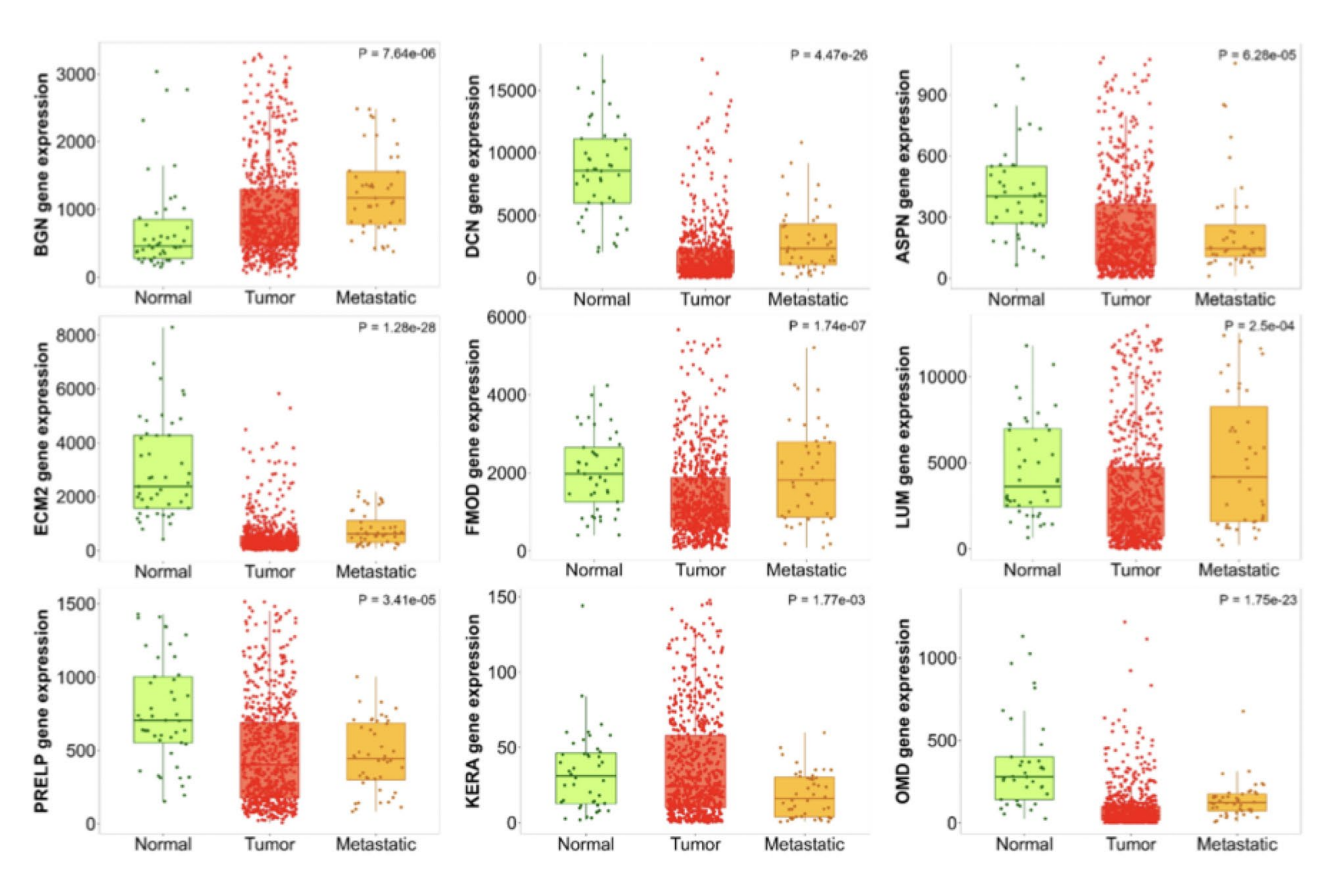
Gene expression in Tumor, Normal and Metastatic tissue. Data obtained from TNM-Plot *(*Bartha and Győrffy 2021*)*

Figure 1 presents the results from the TNM-Plot. It shows that in most of the investigated proteoglycans the median SLRP gene expression was the highest in normal tissue.

The highest gene expression of DCN was in normal tissues, followed by metastatic and tumor tissues. The same order applied to ECM2, FMOD, PRELP and OMD. The highest gene expression of ASPN was found in normal tissue, but the lowest expression was found in metastatic tissue with a highly significant p value. The same order applied to KERA. Exceptions were BGN and LUM. BGN showed the highest gene expression in metastatic tissue, followed by tumor tissue, and the lowest BGN gene expression was presented in normal tissue. LUM had the highest gene expression in metastatic tissue and the lowest in tumor tissue. In most class 1 and class 2 SLRPs the expression was downregulated in tumor and metastatic tissues in comparison to normal tissue samples.

**Fig. 2:**
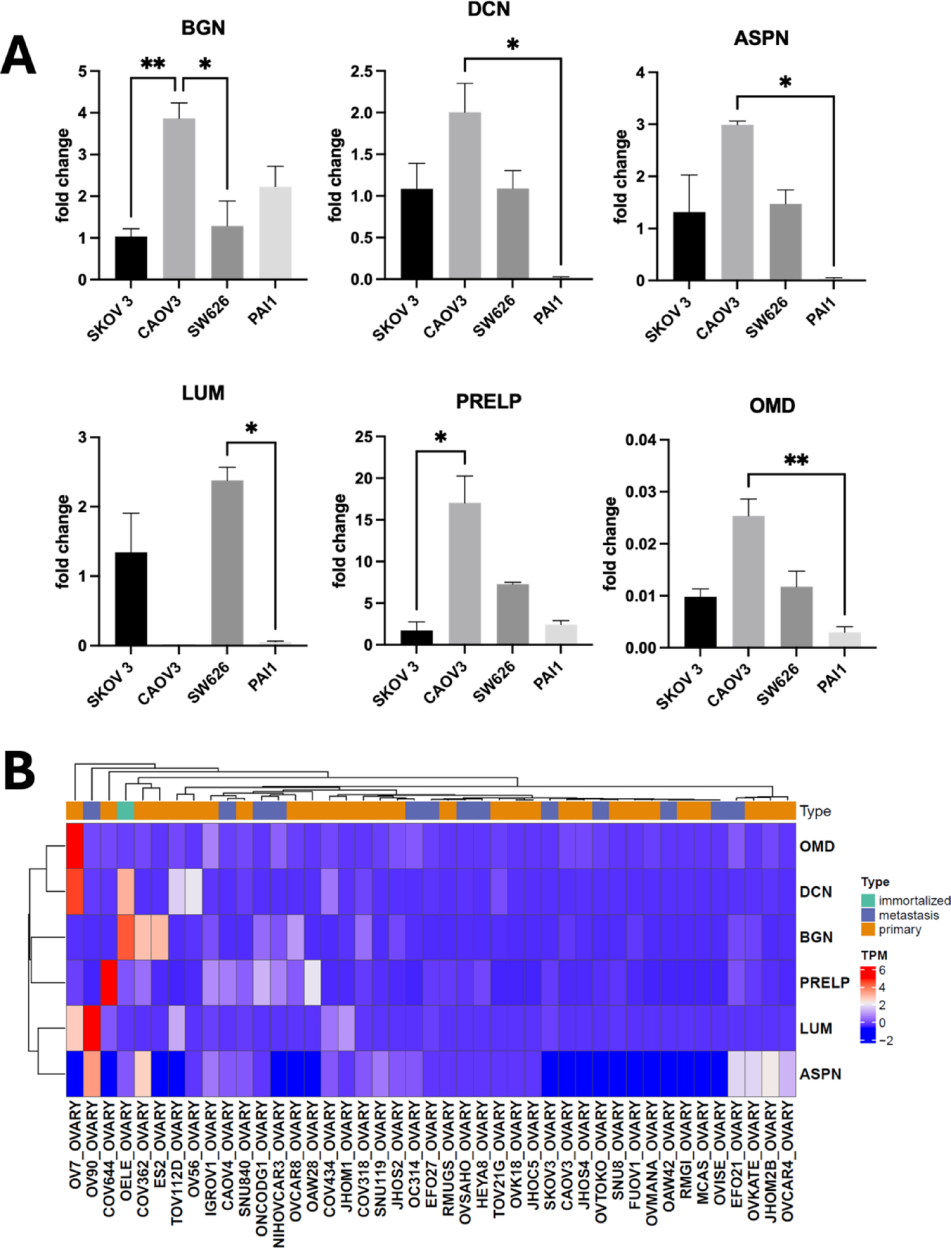
Expression of small leucine-rich proteoglycans (SLRPs) in ovarian cancer cell lines. **A** Relative mRNA expression levels of selected SLRPs (BGN, DCN, ASPN, LUM, PRELP, OMD) in an ovarian cancer panel (SKOV3, CAOV3, SW626, PA1) as determined by quantitative real-time PCR. Gene expression levels are shown as fold change relative to SKOV3. Data represent mean ± standard deviation from three independent experiments. Statistical significance was assessed using one-way ANOVA followed by post hoc Tukey test (*p < 0.05, **p < 0.01). **B** Heatmap of scaled transcript per million (TPM) expression values of SLRP genes across ovarian cancer cell lines from the Cancer Cell Line Encyclopedia (CCLE) dataset (n = 43). Hierarchical clustering was performed based on gene expression patterns. Cell lines are annotated by origin (primary tumor, metastasis, or immortalized). The color scale represents relative expression levels (scaled TPM)

qPCR was used to investigate the expression of six SLRPs in human ovarian cancer cell lines. The selection was based on previous reports of a role of these SLRPs in oncological diseases and diseases affecting biological processes relevant to tumor progression (Appunni et al. 2019; Baghy et al. 2020; Karamanos et al. 2021). The results demonstrated that SLRP expression varies substantially across some ovarian cancer cell lines (Fig. 2A). While certain SLRPs showed increased expression in specific cell lines (e.g. BGN, ASPN, or PRELP in CAOV3), other genes exhibited lower or near baseline expression levels. This variability suggests that individual SLRPs may play distinct roles depending on the molecular background of each cell line, which reflects the heterogeneity of ovarian cancer itself. These disparities in expression patterns may also indicate subtype-specific or context-dependent functions of SLRPs, thereby supporting the hypothesis that their roles in tumor biology are multifaced and potentially tailored to specific tumor environments. To extend the gene expression analysis of ovarian cancer cell lines to a substantially larger panel, we extracted gene expression data on SLRPs of 42 ovarian cancer cell lines and an immortalized healthy ovarian epithelial cell line from the Broad Institute’s Cancer Cell Line Encyclopedia (CCLE) dataset. Overall expression levels of SLRP genes were low across most ovarian cancer cell lines, this is shown in Table 1. A heatmap illustrating the expression patterns of selected SLRP genes across the included cell lines can be found in Fig. 2B. Notably, the included immortalized ovarian cell line exhibited a distinct expression profile, particularly for BGN and DCN. Additionally, certain cell lines such as OV7 and OV90 showed elevated expression of DCN and OMD, as well as ASPN and LUM, respectively. BGN expression was notably higher in the COV362 and ES2 cell lines compared to the others. Although we observed differences in the expression of DCN, OMD, and BGN between primary and metastatic cell lines, these differences were not statistically significant. This may be due to the high variability among the cell lines and the relatively small number of samples.

**Table 1:** Summary of SLRP expression in primary and metastatic ovarian cancer cell lines

Gene ID	Mean in primary	Mean in metastatic	Median in primary	Median in metastatic	Fold Change
DCN	0.45	8.30	0.14	0.31	18.41
ASPN	0.02	0.02	0.01	0.01	0.86
OMD	0.01	0.03	0.01	0.01	2.37
LUM	19.84	10.90	0.30	0.26	0.55
BGN	1.92	5.34	0.21	0.29	2.78
PRELP	0.07	0.08	0.04	0.01	1.09

Expression levels of selected SLRPs are shown as mean and median transcript abundance in primary and metastatic ovarian cancer cell lines based on CCLE data. Fold change represents the ratio of mean expression in metastatic versus primary cell lines

**Fig. 3:**
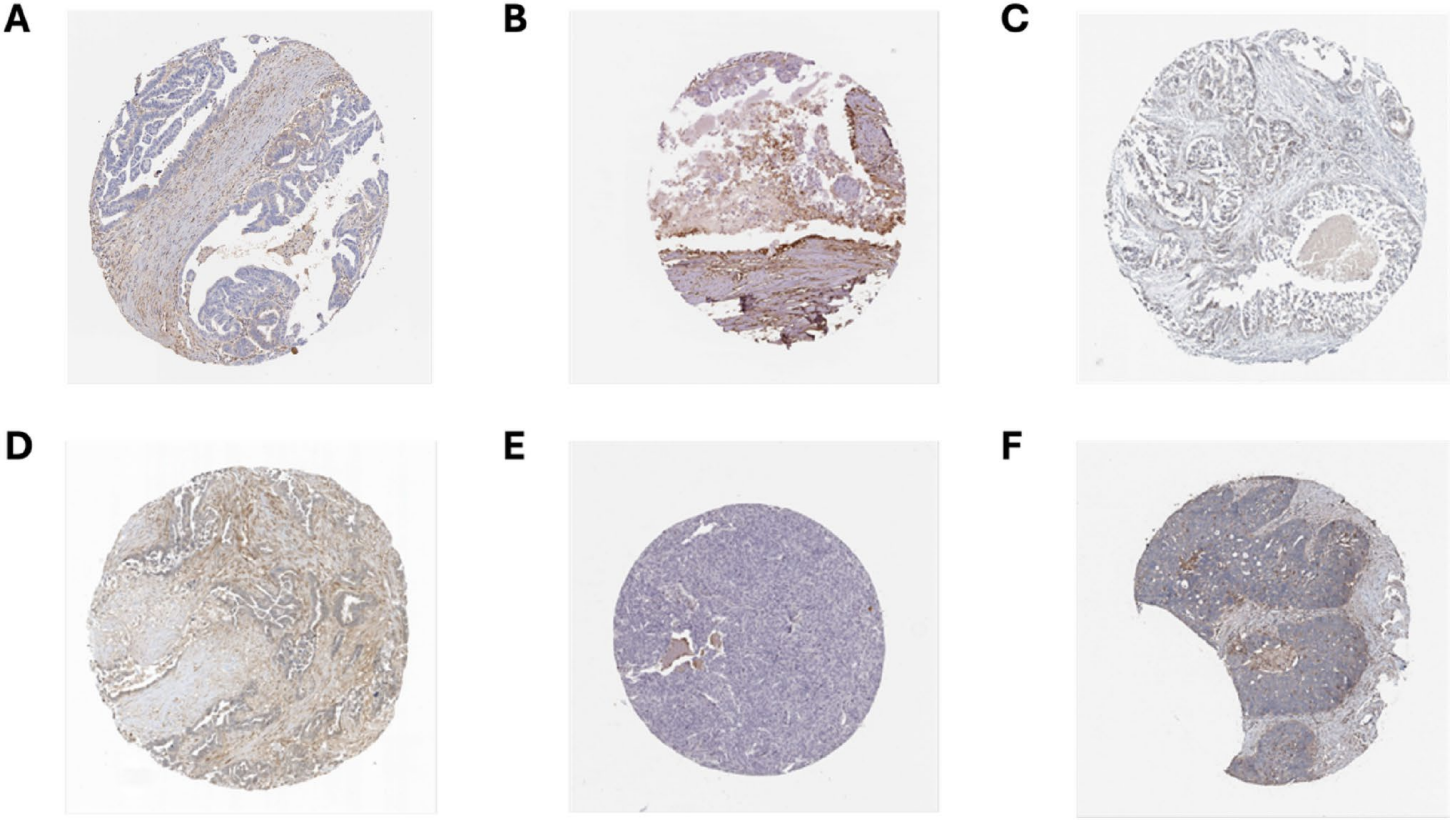
Immunohistochemical staining of SLRPs in serous ovarian carcinoma. Representative immunohistochemical images of small leucine-rich proteoglycans (**A** BGN, **B** DCN, **C** ASPN, **D** ECM2, **E** LUM, **F** KERA) in serous ovarian carcinoma samples obtained from the Human Protein Atlas. Image credit: Human Protein Atlas (Sjöstedt et al. 2020)

To provide spatial context at the protein level, immunohistochemical data from the Human Protein Atlas were analyzed. These data demonstrated detectable expression of several SLRPs in serous ovarian cancer tissue. The staining patterns were predominantly observed in stromal and extracellular matrix compartments, with minimal signal detected in tumor epithelial cells (Fig. 3).

To determine the effect of the expression of SLRPs on the prognosis of serous ovarian cancer a Kaplan–Meier survival analysis was performed. Table 2 presents the results from the Kaplan–Meier Plotter. There were significant results for six SLRPs in progression-free survival, indicating a shorter survival in patients that show higher SLRP levels. Significant results were foundfor BGN, DCN, ASPN, PRELP and OMD. For overall survival there were significant results on a level of significance of *p* < 0.05 for DCN, ASPN and LUM, indicating poorer survival in patients with a high SLRP expression.

**Table 2:** Association of small leucine-rich proteoglycans (SLRPs) expression with survival outcomes in serous ovarian cancer

	Progression free survival (n = 1104)			Overall survival (n = 1207)		
	HR	95% CI	BH-adj. p value	HR	95% CI	BH-adj. p value
BGN	1.27	(1.10–1.47)	**0.0018**	1.16	(1.00–1.35)	0.1008
DCN	1.33	(1.15–1.54)	**0.0004**	1.21	(1.04–1.41)	**0.0293**
ASPN	1.33	(1.15–1.53)	**0.0004**	1.26	(1.08–1.46)	**0.0158**
ECM2	1.15	(1.00–1.33)	0.0694	1.11	(0.95–1.29)	0.2571
FMOD	1.03	(0.89–1.19)	0.7200	0.99	(0.85–1.15)	0.9100
LUM	1.28	(1.11–1.48)	**0.0015**	1.23	(1.05–1.43)	**0.0267**
PRELP*	1.48	(1.20–1.82)	**0.0004**	1.17	(0.94–1.47)	0.2400
KERA	1.10	(0.96–1.27)	0.2025	1.03	(0.89–1.20)	0.7538
OMD	1.37	(1.18–1.58)	**0.0002**	1.10	(0.94–1.28)	0.2957

Hazard ratios (HR), 95% confidence intervals (CI), and Benjamini-Hochberg-adjusted p-values (BH-adjusted p value) for progression-free survival (PFS) and overall survival (OS) are shown for selected SLRPs. Survival analyses were performed using the Kaplan–Meier Plotter database in serous ovarian cancer cohorts (PFS: n = 1104; OS: n = 1207). Hazard ratios > 1 indicate an association between higher gene expression and shorter survival, whereas hazard ratios < 1 indicate an association with longer survival (Győrffy et al. 2012)

The survival curves for progression-free survival and overall survival of the significant results are included in supplementary figures. The largest difference in median progression free survival was seen for PRELP as it was 6 months longer in patients with low expression. The longest median progression free survival was seen in patients with low expression of OMD with a median progression free survival of 20 months.

Lower expression of several SLRPs was associated with improved overall survival in the analyzed datasets. The median overall survival for patients with low expression of DCN was 3.34 months longer compared to patients with high expression of DCN.

Table 3 presents the prognostic significance of the expression of SLRPs on the survival of patients with serous ovarian cancer treated with chemotherapy containing platinum. Most SLRPs showed significant results for the outcome progression free survival. There were significant results for BGN, DCN, ASPN, ECM2, LUM, PRELP and OMD. After multiple testing correction, there were no significant results for overall survival in patients receiving chemotherapy containing platinum. It suggests that there might be a benefit of low expression for survival. Considering median survival, patients with a low expression lived 4.08 months longer without disease progression than patients with a high expression. The biggest difference was observed for OMD. Patients with a low expression of OMD had a median progression free survival of 19.13 months, whereas those with a high expression of OMD showed a progression free survival of 14 months. Table 3 also presents the results for patients with serous ovarian carcinomas that are treated with chemotherapy containing paclitaxel. There were no significant result for any of the SLRPs.

**Table 3:** Association of small leucine-rich proteoglycans (SLRPs) expression with survival outcomes in chemotherapy-defined subgroups

	Chemotherapy containing platinum						Chemotherapy containing paclitaxel					
	Progression free survival (n = 979)			Overall survival (n = 1040)			Progression free survival (n = 229)			Overall survival (n = 220)		
	HR	95% CI	BH-adj. p value	HR	95% CI	BH-adj. p value	HR	95% CI	BH-adj. p value	HR	95% CI	BH-adj. p value
BGN	1.29	(1.12–1.50)	**0.0011**	1.18	(1.00–1.39)	0.0825	0.72	(0.51–1.01)	0.4860	0.97	(0.62–1.53)	0.9100
DCN	1.30	(1.12–1.52)	**0.0010**	1.19	(1.01–1.41)	0.0788	1.01	(0.72–1.41)	0.9700	1.25	(0.79–1.97)	0.6600
ASPN	1.27	(1.09–1.47)	**0.0022**	1.18	(1.00–1.39)	0.0825	0.99	(0.71–1.39)	0.9700	0.68	(0.43–1.07)	0.3880
ECM2	1.17	(1.01–1.36)	**0.03713**	1.11	(0.95–1.32)	0.2571	0.84	(0.60–1.18)	0.5760	0.88	(0.56–1.39)	0.7870
FMOD	1.05	(0.91–1.22)	0.5000	1.04	(0.89–1.23)	0.6000	0.83	(0.59–1.16)	0.5760	0.88	(0.56–1.38)	0.7870
LUM	1.23	(1.06–1.43)	**0.0078**	1.20	(1.02–1.42)	0.0788	0.82	(0.58–1.14)	0.5760	0.77	(0.49–1.21)	0.6667
PRELP*	1.45	(1.18–1.80)	**0.0010**	1.27	(0.99–1.62)	0.0825	1.78	(0.77–4.14)	0.5760	No data available		
KERA	1.06	(0.91–1.23)	0.5000	0.95	(0.81–1.13)	0.6000	0.88	(0.62–1.23)	0.6750	0.85	(0.54–1.33)	0.7520
OMD	1.36	(1.17–1.57)	**0.0005**	1.14	(0.97–1.35)	0.1800	0.90	(0.64–1.26)	0.6943	1.06	(0.67–1.67)	0.9100

Hazard ratios (HR), 95% confidence intervals (CI), and Benjamini-Hochberg-adjusted p-values (BH-adjusted p value) for progression-free survival (PFS) and overall survival (OS) are shown for patients receiving platinum-based chemotherapy (PFS: n = 979; OS: n = 1040) and paclitaxel-containing chemotherapy (PFS: n = 229; OS: n = 220). Hazard ratios > 1 indicate an association between higher gene expression and shorter survival, whereas hazard ratios < 1 indicate an association with longer survival (Győrffy et al. 2012)

**Fig. 4:**
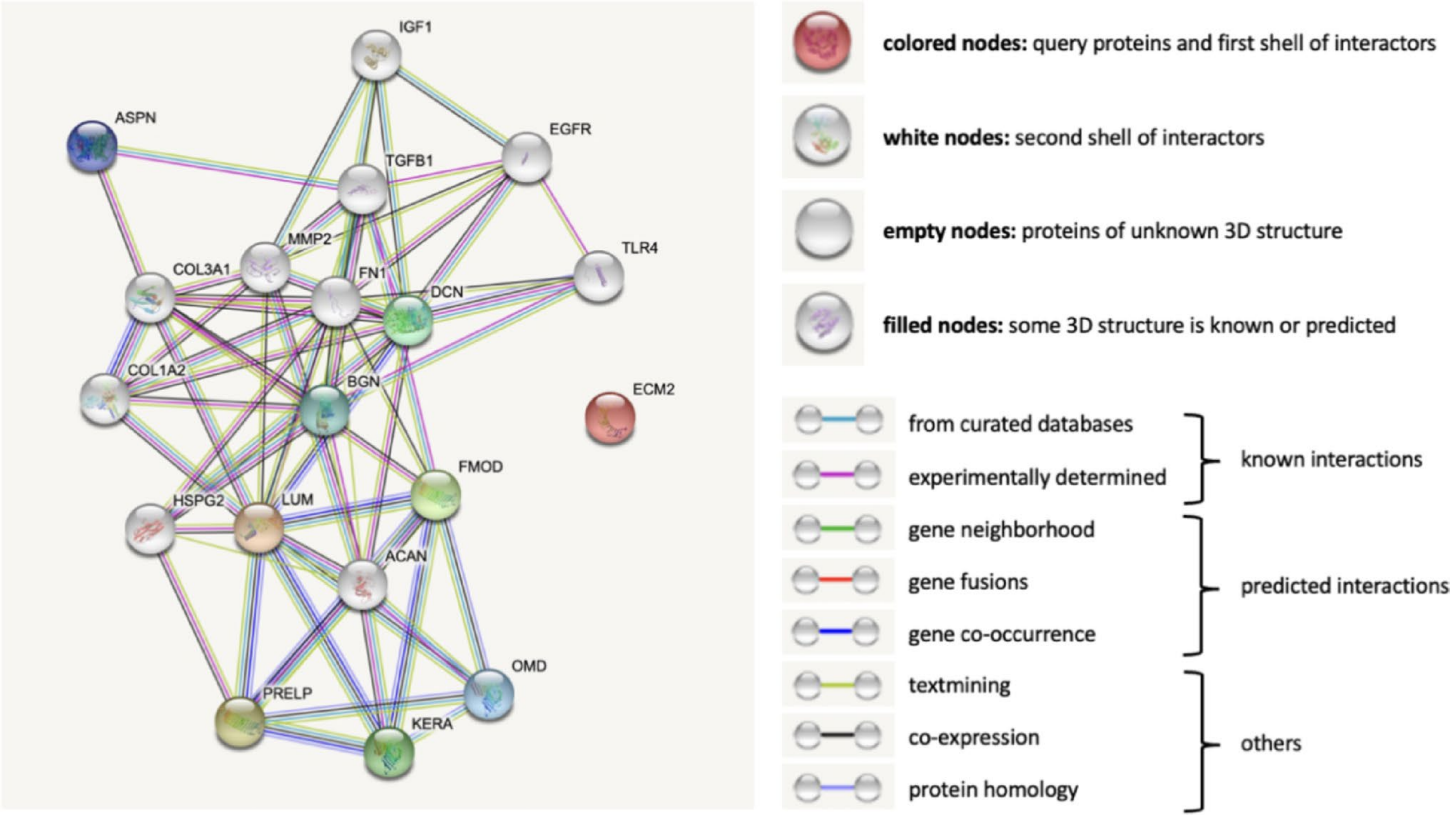
Protein–protein interaction network of SLRPs and associated proteins. Protein–protein interaction network of selected SLRPs generated using the STRING database (Szklarczyk et al. 2021). Coloured nodes represent query proteins and their first shell of interacting partners while white nodes indicate second shell interactors. Edges represent protein–protein interactions derived from curated databases, experimental data, gene co-expression, text mining, and other predictive sources

We finally explored the protein interaction network of SLRPs using STRING analysis (Szklarczyk et al. 2021). As shown in Fig. 4, the interactors identified for the other SLRPs were Insulin-like growth factor 1 (IGF1), Epidermal growth factor receptor (EGFR), Transforming growth factor beta 1 (TGFB1), Collagen Type III Alpha 1 Chain (COL3A1), Matrix Metallopeptidase 2 (MMP2), Fibronectin 1 (FN1), Toll-like receptor 4 (TLR4), Collagen Type I Alpha 2 Chain (COL1A2), Heparan Sulfate Proteoglycan 2 (HSPG2), and Aggrecan (ACAN). The individual interactions can be seen in Fig. 8. Overall, these data suggest that SLRPs potentially form an interaction network with interstitial matrix constituents, matrix degrading enzymes and signaling pathways governing cell proliferation and invasion, with well-documented roles in tumor progression (Karamanos et al. 2021).

## Discussion

Literature showed that SLRPs have a non-uniform role in tumor progression. Some SLRPs are able to inhibit cancer growth, whereas some can enhance tumor growth and lead to rapid progression (Appunni et al. 2019). Our qPCR demonstrated that the expression of SLRPs varies substantially across ovarian carcinoma cell lines of different genetic complexity. Analysis of 42 ovarian cancer cell lines of the CCLE dataset showed that most SLRPs are expressed at relatively low levels in the majority of models, suggesting that SLRP occurrence might be limited to tumor stroma in vivo. Domcke et al. (Domcke et al. 2013) reclassified cell lines by genetic similarity to tumors. Comparing our SLRP expression data to this classification, we noted that ovarian cancer cell lines with an intermediate expression of ASPN and/or a high expression of LUM, PRELP or BGN are classified as high grade serous or possibly high grade serous, respectively. However, a large number of cell lines showed a uniformly low expression of all SLRPs across all categories defined by Domcke et al. (Domcke et al. 2013), therefore, it is difficult to draw general conclusions. The heterogeneity of these cells in terms of TP53-, BRCA1/2-, Myc-, KRAS- and ERBB2- mutations may be an underlying reason for this observation.

In line with our cell line-based gene expression analysis, most SLRPs showed downregulation in tumor tissue vs healthy tissue in clinical samples, as revealed by TNM plot analysis. The dysregulation might play a role in tumorigenesis of serous ovarian cancer. So far there are no studies that show whether a high or low expression of SLRP is advantageous for cancer in general and ovarian cancer in particular, it seems to be different for each SLRP and cancer type (Appunni et al. 2019). With respect to the prognostic impact of our study, the potential biological relevance needs to be separated from the prognostic value. For example, synonymous single nucleotide polymorphisms of genes can have a prognostic value for a given malignant disease, while the amino acid sequence of the affected protein is not altered (Sharma et al. 2019), and it is therefore not intuitively understandable how this polymorphism translates into a change in biological function. Likewise, the suggested prognostic value of the mRNA expression of several SLRPs determined in our study may be of future clinical utility independent of the mechanistic role of the affected protein.

The apparent discrepancy between higher SLRP expression in normal tissue and the association of lower expression with possible improved survival in tumors may reflect RNA vs. protein regulation, subtype differences, and stromal contributions. The TNM Plot database analyzes total RNA levels and does not reflect post-transcriptional regulation. Consequently, mRNA expression may not always directly correlate with functional protein levels. The high SLRP expression in normal ovarian tissue likely reflects contributions from stromal or mesothelial cells. In contrast, the poor prognosis associated with high SLRP expression in tumors may stem from their upregulation within tumor-associated stroma or cancer-associated fibroblasts, where they could actively support tumor progression through ECM remodeling, angiogenesis, or immunosuppressive signaling. SLRPs likely have a context-dependent function (Iozzo and Schaefer 2015). In vivo validation in animal models and spatial transcriptomic profiling would be essential next steps to dissect the functional roles and cellular sources of SLRP expression in serous ovarian tumors.

The immunohistochemical data further support that SLRP expression is not confined to tumor epithelial cells but likely reflects contributions from the tumor microenvironment. This is consistent with the known extracellular matrix-associated nature of SLRPs (Iozzo and Schaefer 2015). Importantly, microenvironmental components such as stromal and extracellular matrix compartments are key regulators of tumor progression and therapeutic response, suggesting that the observed associations may capture biologically relevant tumor-stroma interactions (Winkler et al. 2020; Xu et al. 2025).

This work suggests that the expression of several SLRPs may be associated with progression-free survival in serous ovarian cancer. For DCN, ASPN, and LUM, the overall survival time is was influenced by their expression in our analysis. In association with platinum-based chemotherapy, most SLRPs showed the same results for progression-free survival as in the non treatment specific comparison. Notably, DCN, BGN and their STRING analysis partner FN1 have been suggested to form part of a gene expression signature linked to chemotherapy response in ovarian cancer (Pan et al. 2009). Low expression was correlated with a longer progression-free survival in women treated with chemotherapies containing platinum. For DCN, this finding is partially in contrast to in vitro experiments in human ovarian cancer cell lines that indicated a synergistic anti-tumoral effect of DCN and carboplatin (Nash et al. 1999), which may indicate that the action of DCN is not fully reflected by an in vitro setting that utilizes cancer cells devoid of their microenvironment. In women with serous ovarian cancer that are treated with chemotherapies containing paclitaxel no significant results were found. This may reflect either the drug’s mechanism, which is independent of SLRPs, or differing patient characteristics compared to those treated with platinum.

In contrast to other SLRPs, BGN showed a high expression in tumor and metastatic tissue in the TNM-Plot and low CT-value in the qPCR. Zhao et al. (Zhao et al. 2020) supported these findings of a high BGN expression in ovarian cancer. The Kaplan–Meier Plotter showed that a high mRNA expression is associated with a shorter survival. A reason for that might be that BGN can activate pathways that increase metastasizing (Cong et al. 2021). Thus, a lower expression could potentially limit metastasizing. Similar results were found by Zhao et al. (Zhao et al. 2020). Pan et al. (Pan et al. 2009) found BGN to be overexpressed in chemo-resistant tumor. In line with this, recent functional in vitro studies in ovarian cancer demonstrated that BGN overexpression enhances tumor cell proliferation and migration, whereas its silencing reduces cell viability and migration, supporting a direct role of BGN in promoting tumor aggressiveness (Fang 2025).

The TNM-Plot revealed lower DCN expression in tumor tissue compared to normal tissue, confirmed by high CT values in qPCR. Kaplan–Meier analysis showed significantly longer progression free and overall survival for low expression of DCN. DCN has been suggested to have the ability to stop tumor growth by cell cycle inhibition and anti-angiogenic potential (Appunni et al. 2019). Most studies showed an aggressive tumor growth and progressing disease associated with a low DCN expression (Oda et al. 2012). Other recent experimental data demonstrated that DCN is downregulated in ovarian cancer cells, and that its overexpression suppresses invasion, migration and growth, supporting a tumor-suppressive role at the cellular level in vitro (Sarkar et al. 2025). This is contrary to the results of this work. While DCN expression in tumor cells may exert anti-tumor effects, increased expression in the strima or ECM may reflect microenvironmental remodeling processes that are associated with more aggressive disease. This could be a possible explanaition about the discrepancy of the previous in vitro results and our clinical findings.

Low expression of LUM seems to be beneficial for progression free and overall survival in serous ovarian cancer patients. Likewise, this applied to progression-free survival of patients treated with chemotherapies including platinum. Contrary to those results Iozzo and Schaefer (Iozzo and Schaefer 2015) suggested that LUM is able to reduce tumor progression and migration. Moreover, in a study comparing ovarian cancer growth in LUM wild-type and LUM-deficient mice, Nizet et al. (Nizet et al. 2021) found that LUM inhibits the growth of ovarian cancer cells, suggesting an important role for LUM in the tumor microenvironment. An impact of LUM on collagen fibrillogenesis was identified as an underlying molecular mechanism in this model. On the other hand, LUM was found to be an important factor when it comes to chemotherapy resistance of ovarian cancer which fits the results of this study (Klejewski et al. 2017). In addition, recent spatial proteomic analyses demonstrated that LUM is part of an ECM signature enriched in more advanced tumor regions, together with collagen and other matrix components, suggesting a role in tumor-associated ECM remodeling. These ECM changes have been linked to increased tumor progression, providing further biological context for the observed association between higher LUM expression and poorer survival (Miolo et al. 2025).

According to Simkova et al. (2016), the function of ASPN was different for each cancer type. In ovarian cancer, our data suggested for the first time that ASPN is promoting tumor growth and progression. It seemed to be advantageous to have a low expression of ASPN in ovarian cancer tissue. Another study indicated that ASPN is predominantly expressed by cancer-associated fibroblasts and contributes to tumor progression by promoting cell migration and invasion (Sharma et al. 2024). This supports the biological plausibility of our findings and suggests that ASPN expression may primarily reflect tumor microenvironment interactions rather than tumor cell effects. Our study also demonstrated a prognostic impact of PRELP on ovarian cancer. Notably, in ovarian clear cell carcinoma, misexpression of PRELP was recently noted, but no survival analysis was performed (Dozen et al. 2022). The authors found loss of active histone marks on the loci of the PRELP gene in ovarian clear cell carcinoma patients, and demonstrated an anti-proliferative effect of PRELP in in vitro experiments, suggesting a more general tumor suppressor role for PRELP in ovarian cancer. While we also demonstrated a prognostic value for ECM2 and OMD in ovarian cancer, no studies on these SLRPs have been published so far, indicating a need for further research.

Out of the ten interactors that were suggested by the STRING database only four are known to be important factors in cancer pathogenesis and metastasis. They include TGFB1, EGFR, MMP2 and FN1. TGFB1 plays a role in proliferation, differentiation and cell growth, all of which are important factors for malignant tumors. In ovarian cancers TGFB1 is a strong inducer of epithelial-to-mesenchymal transition and is involved in the invasive behaviour of the tumor (Basu et al. 2015). TGFB1 interacted with ASPN, DCN, FMOD and BGN. Most of those SLRPs have shown a significant effect on the survival of serous ovarian cancer in our analysis.

There are several pathways in which EGFR could have an influence on cancer. One of them is, for example, the EGF-EGFR signaling pathway which plays a role in metastasis (Ji et al. 2022). According to Zhao et al. (2020), a high activation of EGFR enhanced the resistance against cisplatin suggesting that repression of EGFR leads to a better function of therapy with cisplatin and therefore probably a better prognosis for the patients. EGFR was mainly found to have interactions with DCN which had significant differences in progession-free survival when comparing high and low expression in serous ovarian cancer patients treated with chemotherapy containing platinum. Hu et al. (2021) showed that DCN is able to inhibit the EGFR pathway in breast cancer. Thus, it can be assumed that these are interactions that might be relevant to the effect of chemotherapies and the prognosis of cancer patients. MMP2 is involved in metastasis and proliferation of ovarian cancers. In accordance with its molecular function as an ECM-remodeling enzyme, modulation of MMP2 expression might result in reduced invasive growth of ovarian carcinoma cells (Wang et al. 2018). FN1 has been suggested to play a role in metastasis by influencing cell adhesion and migration (Liang et al. 2020).

A major limitation of this study is that the survival analyses are based on univariable Kaplan–Meier estimates derived from publicly available datasets using the Kaplan–Meier Plotter tool. Thus we did not include an adjustment for important clinical covariates such as age, tumor stage, residual disease, or BRCA/HRD status. Therefore, the observed associations may be influenced by confounding factors and should be interpreted with caution.

In order to provide further validation, it is imperative to conduct in vivo studies using ovarian cancer mouse models in the future. While the present data are based on clinical samples and in vitro experiments, animal models can provide mechanistic insights into the role of SLRPs in tumor progression and treatment response. Previous studies have yielded conflicting results, contingent upon the SLRPs and the tumor context.

Our findings suggestes that lower expression of several SLRPs correlate with significantly improved survival in serous ovarian cancer. These findings raise the hypothesis that SLRPs may represent potential therapeutic targets, which requires further functional validation. To the best of our knowledge, no clinical trials are currently evaluating SLRP inhibitors in ovarian cancer nor in other disease contexts. Further mechanistic studies are required to clarify the pathways through which SLRPs exert their influence on tumor progression and patient survival. This would help determine whether SLRP expression is causative in chemoresistance or progression, or merely a correlated marker. If preclinical experiments in vivo prove successful, development of targeted inhibitors or therapeutic peptides could pave the way toward novel personalized therapies for serous ovarian cancer patients.

## Conclusion

This study identifies several class 1 and class 2 SLRPs as potential prognostic markers in serous ovarian cancer. Lower expression of multiple SLRPs was consistently associated with improved progression-free and overall survival in serous ovarian cancer patients in general and in those receiving chemotherapy containing platinum. These findings highlight a potential role of SLRPs in ovarian cancer progression and could advance future biomarker development. Further validation in clinically well-annotated cohorts and functional studies will be essential to confirm their prognostic and therapeutic relevance.

## Supplementary Information

Below is the link to the electronic supplementary material.

Supplementary Material 1.

## Data Availability

Data using the TNM Plot tool were assembled from public datasets including gene arrays from the Gene Expression Omnibus of the National Center for Biotechnology Information (NCBI-GEO) or RNA-seq from The Cancer Genome Atlas (TCGA), Therapeutically Applicable Research to Generate Effective Treatments (TARGET), and The Genotype-Tissue Expression (GTEx) repositories, and have been described in the respective original publication (Bartha, Á., & Győrffy, B. (2021). TNMplot. com: a web tool for the comparison of gene expression in normal, tumor and metastatic tissues. International journal of molecular sciences, 22(5), 2622. 10.3390/ijms22052622). Data from the KMPlot tool were assembled from eight public datasets and are described in the original publication (Győrffy, B., Lánczky, A., & Szállási, Z. (2012). Implementing an online tool for genome-wide validation of survival-associated biomarkers in ovarian-cancer using microarray data from 1287 patients. Endocrine-related cancer, 19(2), 197–208. 10.1530/ERC-11-0329). Data from the STRING tool utilize more than 10 public databases, as defined in the original publication (Szklarczyk, D., Gable, A. L., Nastou, K. C., Lyon, D., Kirsch, R., Pyysalo, S., … & von Mering, C. (2021). The STRING database in 2021: customizable protein–protein networks, and functional characterization of user-uploaded gene/measurement sets. Nucleic acids research, 49(D1), D605-D612. 10.1093/nar/gkaa1074). Raw data of the individual studies are either available in the cited public repositories or from the authors of the original publications upon request.

## Abbreviations

SLRP: Small leucine rich proteoglycans
HER2: Human epidermal growth factor receptor 2
BGN: Biglycan
CCLE: Cancer Cell Line Encyclopedia
DCN: Decorin
ASPN: Asporin
ECM2: Extracellular matrix protein 2
ECMX: Extracellular matrix protein X
FMOD: Fibromodulin
LUM: Lumican
PRELP: Proline and arginine rich end leucine rich repeat protein
KERA: Keratocan
OMD: Osteomodulin
ATCC: American Type Culture Collection
PCR: Polymerase Chain Reaction
IGF1: Insulin-like growth factor
EGFR: Epidermal growth factor receptor
TGFB1: Transforming growth factor beta 1
COL3A1: Collagen Type III Alpha 1 Chain
MMP2: Matrix Metallopeptidase 2
FN1: Fibronectin 1
TLR4: Toll-like receptor 4
COL1A2: Collagen Type I Alpha 2 Chain
HSPG2: Heparan Sulfate Proteoglycan 2
ACAN: Aggrecan
