# Supplementary material for: Prognostic influence of small leucine-rich proteoglycans on serous ovarian cancer
Source: J Cancer Res Clin Oncol. 2026 Jun 7;152(6):117. doi: 10.1007/s00432-026-06529-2 (PMC13249986; doi:10.1007/s00432-026-06529-2)
Supplement: Supplementary file 1 — Supplementary Material 1. [file 432_2026_6529_MOESM1_ESM.docx]

**Supplementary Table S1: Primer sequences used for qPCR analysis of SLRPs.** Forward and reverse primer sequences for selected SLRPs analyzed in this study are listed. Primers were used for amplification of target genes in ovarian cancer cell lines.

| **SLRP** | **Primer sequences** |
| --- | --- |
| BGN | Forward: *TTGAACCTGGAGCCTTCGATGG* |
|  | Reverse: *TTGGAGTAGCGAAGCAGGTCCT* |
| DCN | Forward: *GCTCTCCTACATCCGCATTGCT* |
|  | Reverse: *GTCCTTTCAGGCTAGCTGCATC* |
| ASPN | Forward: *CCACCAACTTTATTGGAGCTTCAC* |
|  | Reverse: *CGTGGTATGTTAGCAAGACTCCC* |
| LUM | Forward: *AACATACCAACTGTCAATGAAAACC* |
|  | Reverse: *TGCCATCCAAACGCAAATGCTTG* |
| PRELP | Forward: *CGCCATCAACAACAGGCTGGAA* |
|  | Reverse: *TCCGAGGAGAAGTCATGGAACG* |
| OMD | Forward: *GTAGAACTCAGTGTTGGACACAAC* |
|  | Reverse: *GGTCAATAGAAGGACACATCACTG* |

**Supplementary Table S2: Raw Ct values of SLRPs in ovarian cancer cell lines.** Ct values of selected SLRPs and the reference gene (Actin) are shown for ovarian cancer cell lines. qPCR was performed in three independent biological replicated for each cell line. Values represent raw Ct measurements used for subsequent normalization and calculation of relative gene expression.

|  | **Actin** | BGN | DCN | ASPN | LUM | PRELP | OMD |
| --- | --- | --- | --- | --- | --- | --- | --- |
| SKOV3 (1) | 11.15 | 25.73 | 26.80 | 30.08 | 26.90 | 30.39 | 0 |
| SKOV3 (2) | 10.53 | 24.97 | 26.88 | 29.60 | 23.95 | 32.17 | 30.37 |
| SKOV 3 (3) | 11.11 | 24.89 | 26.02 | 27.93 | 23.96 | 28.71 | 31.40 |
| CAOV3 (1) | 11.77 | 24.33 | 26.88 | 28.53 | 0 | 27.49 | 30.84 |
| CAOV3 (2) | 12.50 | 24.58 | 27.18 | 29.17 | 0 | 27.44 | 30.91 |
| CAOV3 (3) | 12.87 | 25.22 | 27.11 | 29.52 | 32.50 | 28.56 | 31.50 |
| SW626 (1) | 11.55 | 24.67 | 26.80 | 28.93 | 24.20 | 28.15 | 31.22 |
| SW626 (2) | 10.80 | 24.50 | 26.06 | 29.14 | 23.44 | 27.37 | 30.11 |
| SW626 (3) | 10.50 | 27.40 | 26.73 | 28.09 | 23.51 | 27.24 | 31.24 |
| PA1 (1) | 9.48 | 23.38 | 32.52 | 33.35 | 28.20 | 28.20 | 31.79 |
| PA1 (2) | 9.92 | 22.89 | 31.33 | 32.32 | 28.47 | 28.18 | 30.91 |
| PA1 (3) | 10.11 | 22.82 | 30.65 | 32.99 | 27.78 | 27.89 | 32.57 |
|  |  |  |  |  |  |  |  |

**Supplementary Table S3: Human Protein Atlas image sources for immunohistochemical analysis of SLRPs.** Direct links tot he Human Protein Atlas entries and corresponding immunohistochemical images for selected SLRPs in serous ovarian cancer tissue are provided (Sjöstedt et al. 2020). These sources were used for qualitative assessment of protein expression and spatial localization withing tumor samples.

| **SLRP** | **Direct links** |
| --- | --- |
| BGN | https://www.proteinatlas.org/ENSG00000182492-BGN/pathology/ovarian+cancer |
| DCN | https://www.proteinatlas.org/ENSG00000011465-DCN/pathology/ovarian+cancer |
| ASPN | https://www.proteinatlas.org/ENSG00000106819-ASPN/pathology/ovarian+cancer |
| ECM2 | https://www.proteinatlas.org/ENSG00000106823-ECM2/pathology/ovarian+cancer |
| LUM | https://www.proteinatlas.org/ENSG00000139329-LUM/pathology/ovarian+cancer |
| KERA | https://www.proteinatlas.org/ENSG00000139330-KERA/pathology/ovarian+cancer |


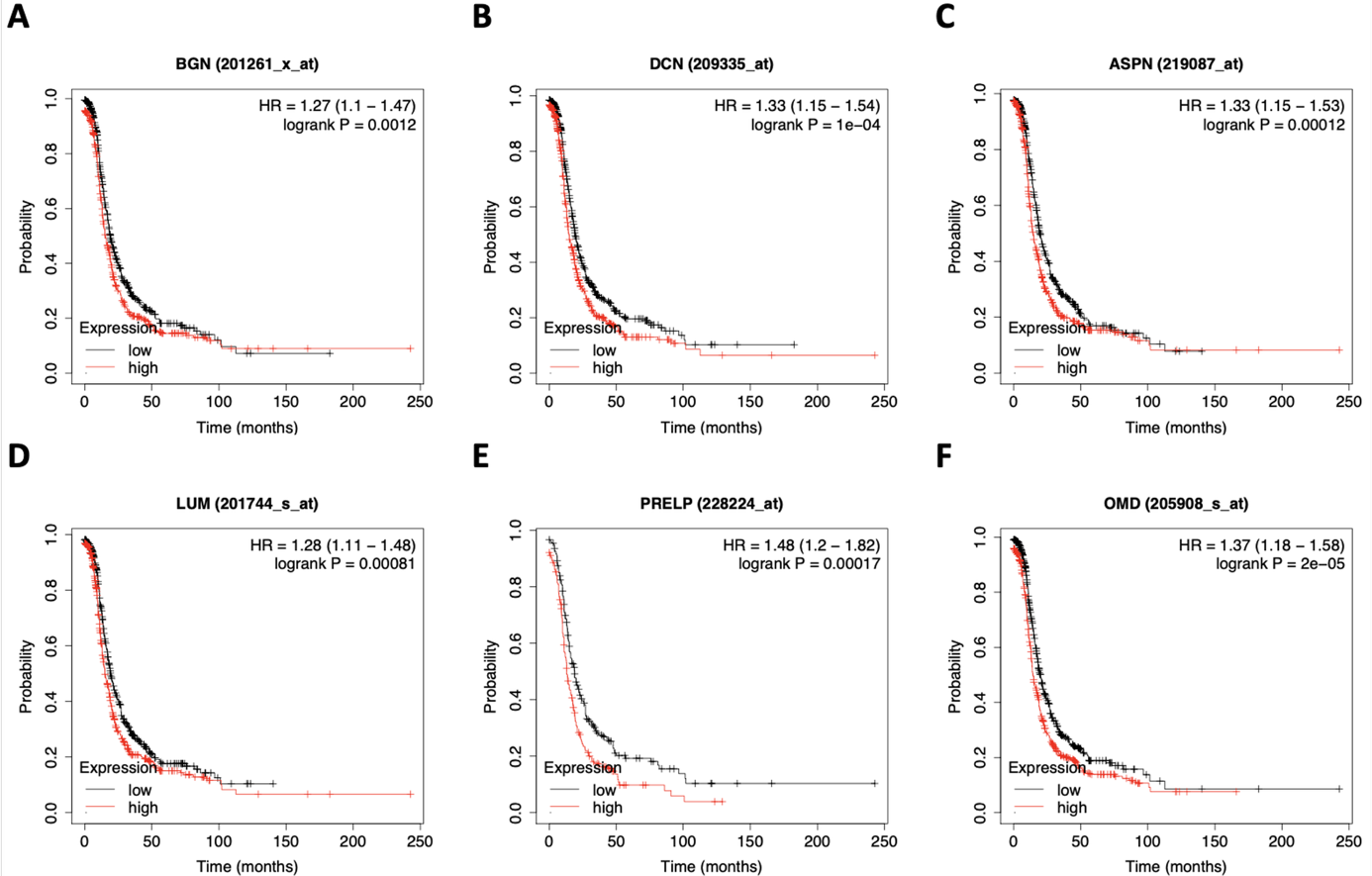


**Supplementary Figure S1: Association between SLRP expression and progression-free survival in serous ovarian cancer.** Kaplan-Meier survival curves showing progression-free survival of serous ovarian cancer patients stratified by high versus low expression of selected SLRPs. Survival analyses were performed using the Kaplan-Meier-Plotter database (Györffy et al., 2012). Patients were divided into high and low expression groups based on the median expression of each gene. Hazard ratios (HR) with 95% confidence intervals and log-rank p-values are indicated in each panel.


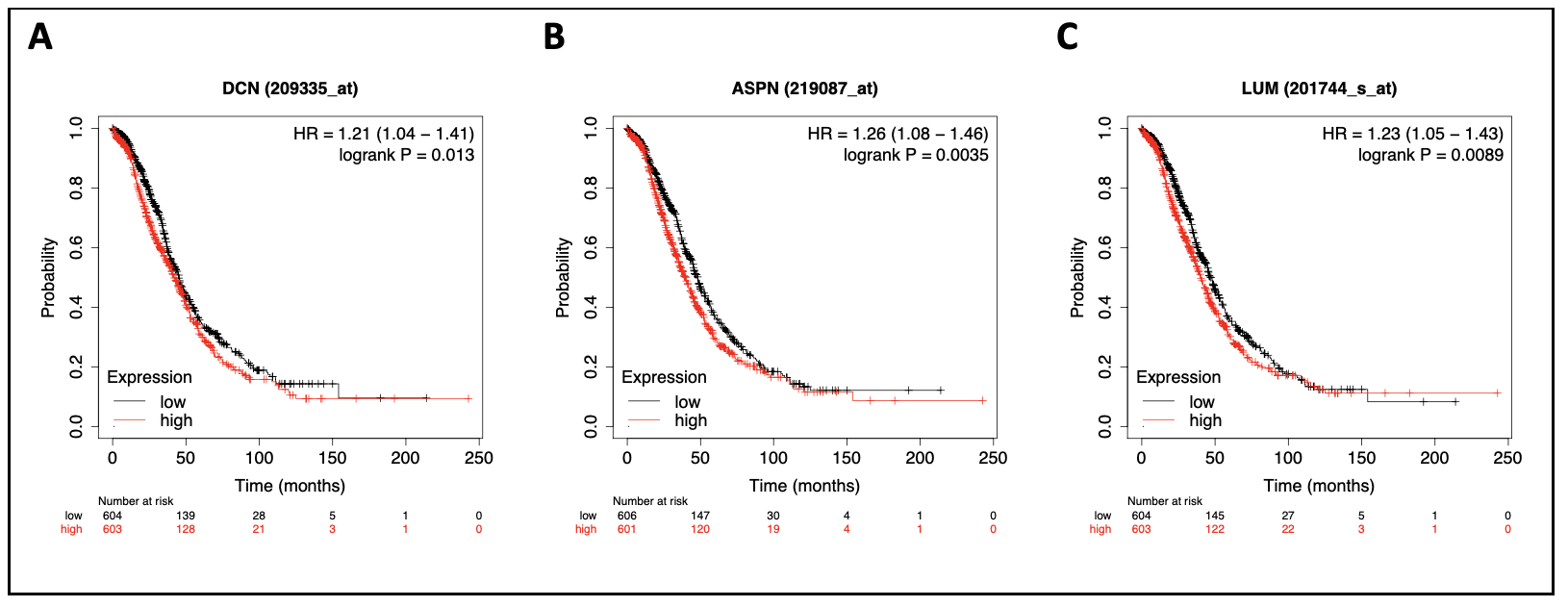


**Supplementary Figure S2: Association between SLRP expression and overall survival in serous ovarian cancer.** Kaplan-Meier survival curves showing overall survival of serous ovarian cancer patients stratified by high versus low expression of selected SLRPs. Survival analyses were performed using the Kaplan-Meier-Plotter database (Györffy et al., 2012). Patients were divided into high and low expression groups based on the median expression of each gene. Hazard ratios (HR) with 95% confidence intervals and log-rank p-values are indicated in each panel.


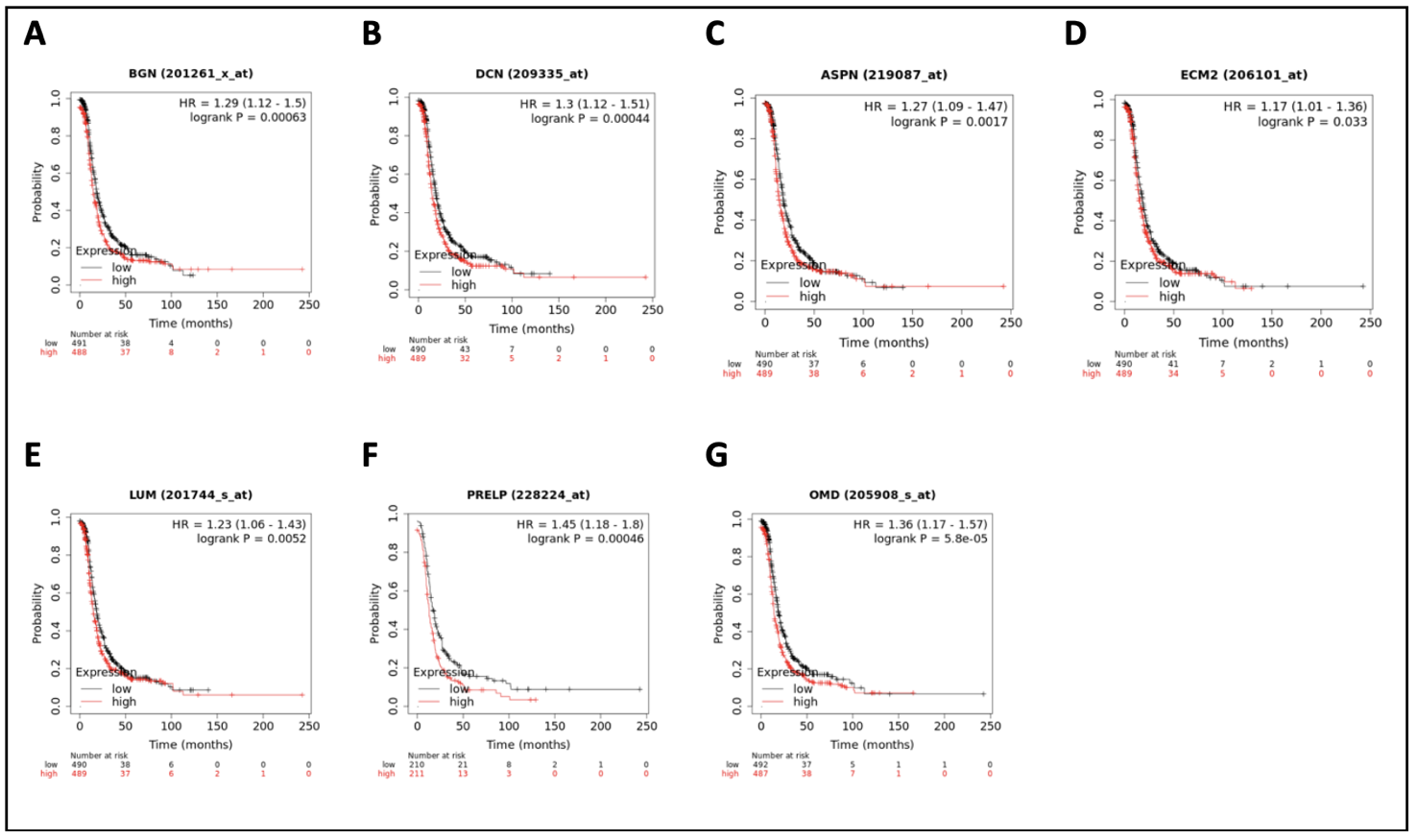

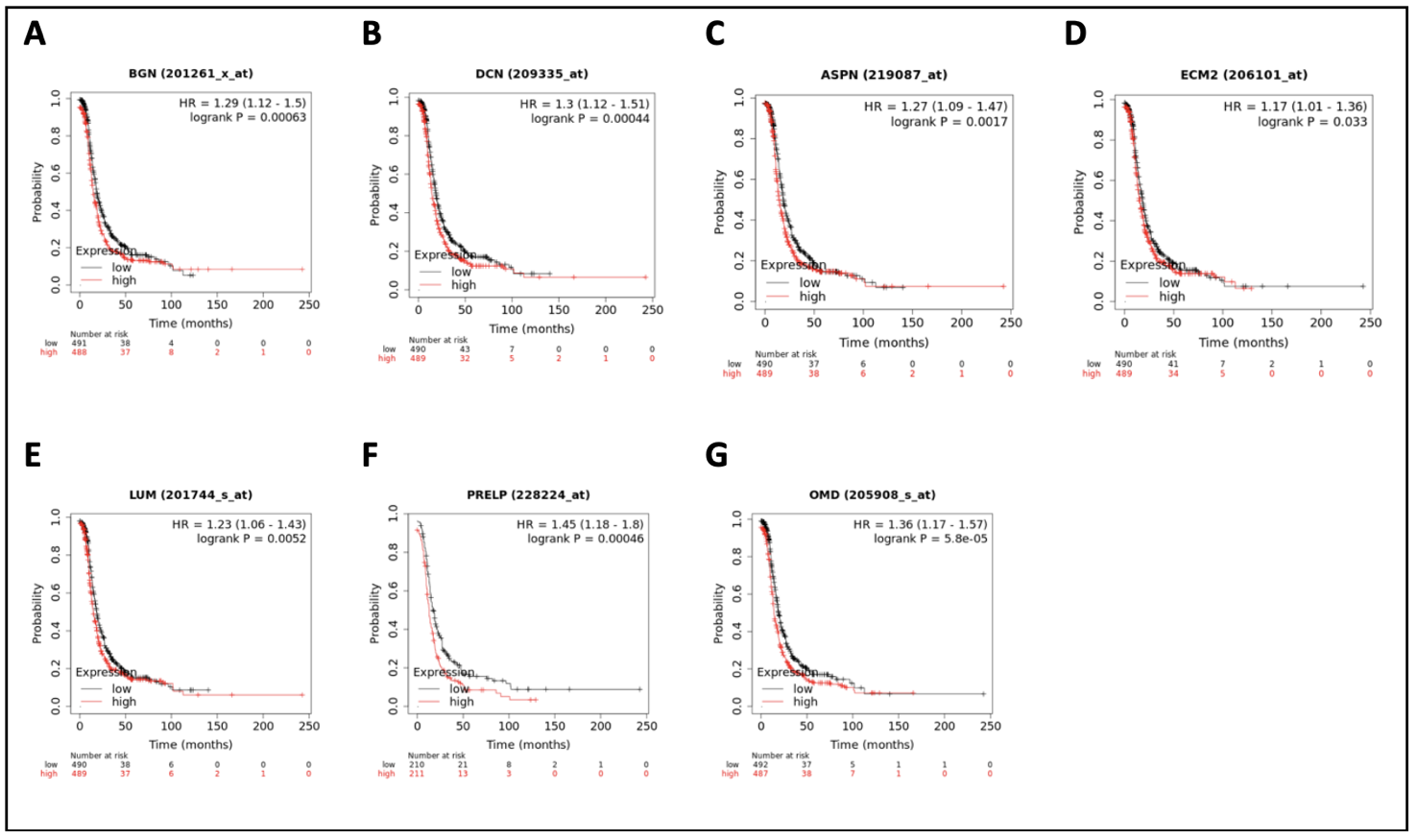


**Supplementary Figure S3: Association between SLRP expression and progression-free survival in patients with serous ovarian cancer treated with chemotherapy containing platinum.** Kaplan-Meier survival curves showing progression-free survival of serous ovarian cancer patients treated with chemotherapy containing platinum stratified by high versus low expression of selected SLRPs. Survival analyses were performed using the Kaplan-Meier-Plotter database (Györffy et al., 2012). Patients were divided into high and low expression groups based on the median expression of each gene. Hazard ratios (HR) with 95% confidence intervals and log-rank p-values are indicated in each panel.
